# Paradigmatic Relations Interact During the Production of Complex Words: Evidence From Variable Plurals in Dutch

**DOI:** 10.3389/fpsyg.2021.720017

**Published:** 2021-09-01

**Authors:** Tim Zee, Louis ten Bosch, Ingo Plag, Mirjam Ernestus

**Affiliations:** ^1^Centre for Language Studies, Radboud University, Nijmegen, Netherlands; ^2^Department of English Language and Linguistics, Heinrich Heine University, Düsseldorf, Germany

**Keywords:** morphology, phonetics, paradigms, reduction, inflection, Dutch, plural, variation

## Abstract

A growing body of work in psycholinguistics suggests that morphological relations between word forms affect the processing of complex words. Previous studies have usually focused on a particular type of paradigmatic relation, for example the relation between paradigm members, or the relation between alternative forms filling a particular paradigm cell. However, potential interactions between different types of paradigmatic relations have remained relatively unexplored. This paper presents two corpus studies of variable plurals in Dutch to test hypotheses about potentially interacting paradigmatic effects. The first study shows that generalization across noun paradigms predicts the distribution of plural variants, and that this effect is diminished for paradigms in which the plural variants are more likely to have a strong representation in the mental lexicon. The second study demonstrates that the pronunciation of a target plural variant is affected by coactivation of the alternative variant, resulting in shorter segmental durations. This effect is dependent on the representational strength of the alternative plural variant. In sum, by exploring interactions between different types of paradigmatic relations, this paper provides evidence that storage of morphologically complex words may affect the role of generalization and coactivation during production.

## 1. Introduction

Most psycholinguistic accounts of lexical processing agree that the comprehension and production of a word form can be affected by its morphological relations with other word forms (see, for example, the recent overview in Arndt-Lappe and Ernestus, 2020). In very general terms, two words can be seen as morphologically related if they share phonological features that also reflect a similarity in meaning. Broadly, two types of morphological relations can be distinguished: relations between words that share a base (e.g., *burn* and *burned*) and relations between words with shared inflectional or derivational exponence (e.g., *burned* and *cared*). In this paper, we will refer to the former as relations within paradigms and to the latter as relations between paradigms. We will make a further distinction between two types of within-paradigm relations: those between the base and a complex form (e.g., *burn* and *burned*), and those between two alternative forms (e.g., *burned* and *burnt*). Previous psycholinguistic studies on morphological relations have mostly focused on how the different relation types individually affect word processing (e.g., Ernestus and Baayen, 2003 for between-paradigm relations; Hay, 2001 for base-complex relations; Cohen, 2015 for relations between alternatives). Potential interactions between these different types of paradigmatic relations have remained relatively unexplored (but see Milin et al., 2009). In the current research, we will use Dutch plurals to investigate the potentially interacting effects of between- and within-paradigm relations. In doing so, we aim to contribute to a more complete understanding of the mechanisms of generalization, storage and coactivation that are involved in the processing of complex words.

Most Dutch plural nouns are inflected for number by suffixing the singular base form with either of the two regular suffixes *-en* and *-s*. In addition, a few plurals are formed with irregular suffixes such as *-eren* or *-a*. As noted by dictionaries (e.g., Van Dale, 2020) and textbooks on Dutch morphology (e.g., de Haas and Trommelen, 1993), for certain nouns more than one suffix is acceptable: *artikel* “article” can be inflected as both *artikels* and *artikelen*, and both *keuzes* and *keuzen* are acceptable plurals of *keuze* “choice.” Although some of this variability can be attributed to differences in modality (Kürschner, 2009), register (Baayen et al., 2002) and dialect (Goeman et al., 2005), different plural forms of the same noun can be found in a single utterance, see (1) which was taken from Wilde Haren De Podcast (2019).

(1) de **piramides** van Gizeh, hè, de drie bekende ‘the pyramids of Giza, right, the three famous **piramiden**
  pyramids’

Baayen et al. (2002) argue that this type of variation occurs when the factors that govern the allomorphy within the Dutch plural system are inconclusive. For instance, most accounts of the Dutch plural agree that the distribution of *-en*, pronounced /ə(n)/, vs. *-s*, pronounced /s/, seems to reflect a prosodic preference for a word-final disyllabic trochee. As a result, most nouns with an unstressed final syllable, e.g., *bakker* /ˈbɑkər/, are pluralized with *-s*, whereas most nouns ending in a stressed syllable are pluralized with *-en*, e.g., *dier* /ˈdir/. However, if a singular noun already ends in schwa, e.g., *piramide* /ˌpiraˈmidə/, the *-en* suffix is simplified to *-n*, such that adding either suffix would result in a word-final trochee and, as a result, an acceptable plural (Kürschner, 2009). Variation may also occur when two factors are in conflict. For instance, the phonological generalization that nouns ending in stressed vowels have the plural suffix *-s* sometimes conflicts with the preference for a trochee. This may explain the variation in the plural of the noun *individu* /ˌɪndiviˈdy/: *individu's* and *individuen*. In sum, previous discussions of Dutch variable plurals suggest that two alternative forms may exist as a consequence of the application of non-deterministic phonological generalizations. However, we will argue that storage and coactivation mechanisms might also be expected to affect the production of variable plurals, given the different paradigmatic relations that apply to variable plurals. As such, Dutch variable plurals provide an excellent opportunity to investigate how different types of paradigmatic relations interact.

### 1.1. Paradigmatic Relations

Dutch variable plurals are a suitable phenomenon to illustrate how between-paradigm relations may affect morphological processing. The morpho-phonological patterns that, according to Baayen et al. (2002), govern both the distribution of invariable and variable Dutch plurals can be seen as generalizations among noun paradigms. In fact, these between-paradigm generalizations can be explicitly modeled using the mechanism of analogy. For instance, in order to produce the plural form of *vampier* “vampire,” generalization by analogy relies on morpho-phonological similarities to singular base forms from other paradigms such as *pionier* “pioneer” and generalizes their plural forms, i.e., *pioniers*, to the original base form, resulting in *vampiers*. An advantage of such an analogical approach is that the production of variation is built-in: the plural of *vampier* can also be generalized from the *papier-papieren* “paper(s)” paradigm, resulting in *vampieren*, which is also an acceptable form. Previous work has shown that computational analogical models accurately predict the variation observed for various phonological and morphological phenomena, and affix choice in particular (e.g., Krott et al., 2001; Wulf, 2002; Ernestus and Baayen, 2003; Keuleers et al., 2007; Arndt-Lappe, 2014). Although analogical models elegantly predict the occurrence of many affixed forms that would be classified as exceptions in categorical rule-based models, analogical mechanisms are not completely successful in their predictions either. The model implemented by Keuleers et al. (2007) shows that inaccurate predictions also exist for Dutch plurals. Although this model improved on the performance of a deterministic rule-based model, it still attributed the wrong allomorph to around 9% of the plural forms they considered. This suggests that not every Dutch plural form can be predicted from between-paradigm relations.

It has been argued that the influence of between-paradigm relations on lexical processing is limited for word forms with high token frequencies (e.g., Bybee, 1995). The reasoning behind this claim is that repeated exposure to a word form results in a strong representation which is easier to access directly, compared to weaker representations of infrequent word forms, which may be easier to process by generalization from related word forms (e.g., Divjak and Caldwell-Harris, 2019). Such storage effects might affect the distribution of morphological structure in a language. For example, Bybee (1995) argues that the irregular past tense in English tends to occur in frequent verbs because their strong representations have resisted the generalization from phonologically similar regular past tense forms (see also Cuskley et al., 2014). This suggests that absolute token frequency is a measure of representational strength. However, some studies (Hay, 2001, 2007; Blumenthal-Dramé, 2012) have claimed that representational strength of complex forms is best measured as the token frequency of the complex word relative to its base word. Hay (2001) observes that models of lexical processing which incorporate both computation and whole-word access involve some type of competition between whole-word representations and representations of the base (e.g., Baayen et al., 1997b). It follows, according to Hay (2001), that relative frequency between these forms, rather than absolute frequency of the complex form, is a better predictor of the degree to which complex representations are accessed directly in lexical processing. Psycholinguistic evidence for this base-complex frequency relation has come from studies on derived words (e.g., Hay, 2001, 2007; Blumenthal-Dramé, 2012) in addition to findings from plural inflection (e.g., Baayen et al., 1997a,b, 2003; New et al., 2004; Biedermann et al., 2013; Beyersmann et al., 2015). For instance, Baayen et al. (1997b) showed that Dutch singular nouns are processed faster than their plural inflections but only if they are singular-dominant, i.e., if the singular forms are more frequent than the corresponding plurals. These findings have led researchers to posit that processing of singular-dominant plurals often requires computation based on the singular, resulting in slower and less accurate processing (Beyersmann et al., 2015). Conversely, in a picture naming study, Baayen et al. (2008) concluded that shorter production latencies for Dutch plural-dominant plurals may reflect that their production is less dependent on analogical generalization. In sum, the base-complex frequency relation has been argued to mediate between distinct processing mechanisms: direct activation of a representation vs. some form of generalization, be it through rules or analogy.

Within-paradigm frequency relations have also been found to affect the phonetic realization of morphologically complex words. For instance, Cohen (2014) found that when speakers read aloud sentences like *The choir for the church services seems nervous*, the verb agreement suffix *-s* was longer if the 3rd person singular form (e.g., *seems*) was frequent compared to the uninflected form (e.g., *seem*). Various studies have found similar phonetic enhancement of complex words with a higher frequency relative to one or more members of their paradigm (Kuperman et al., 2007; Schuppler et al., 2012; Bell et al., 2020; Tomaschek et al., 2021b, but see Hanique and Ernestus, 2012). This so-called *paradigmatic enhancement* effect has been argued to occur when the choice between multiple paradigm members is probabilistic (Kuperman et al., 2007). However, studies vary considerably with regard to the paradigm members they deem to contribute to this effect. In the current research, we will follow Cohen (2014) and Cohen (2015) by only considering the *paradigmatic enhancement* effect associated with the frequency relation between paradigm members that are allowed by the syntactic context and that result in a very similar meaning. We can illustrate such *paradigmatic alternatives* using Dutch variable plurals: in *De drie bekende piramides/piramiden* “the three famous pyramids,” both plurals are allowed by the syntactic context and the resulting semantics are very similar (if they differ at all). If paradigmatic enhancement applies to Dutch variable plurals we would expect the frequency ratio between plural variants to affect their pronunciation.

Paradigmatic enhancement can be formulated in terms of probability: words with a higher paradigmatic probability have more enhanced pronunciations. In that light, paradigmatic enhancement is a surprising effect, given many previous studies which show that increased probability of a linguistic structure generally results in reduced pronunciations. For instance, it has been shown that contextually probable segments (e.g., van Son and Pols, 2003), syllables (e.g., Aylett and Turk, 2006), and words (e.g., Bell et al., 2009) are reduced in terms of duration and/or spectral qualities. Moreover, there is even some evidence that increased probability of a complex word relative to its base results in reduced pronunciation (Hay, 2001). This tendency to reduce predictable units can be explained from a communicative perspective if we assume that speakers reduce elements that contribute less to listener comprehension (e.g., Aylett and Turk, 2004). In addition to this listener-oriented account, an alternative, potentially better supported (Bell et al., 2009; Ernestus, 2014), speaker-driven account of reduction has been proposed. In such an account, the reduction of predictable words can be explained using two mechanisms that are relevant to the current study. Firstly, it has been proposed that representations of more predictable words are easier to access, which allows for faster articulation (e.g., Bell et al., 2009). Secondly, the reduction of high probability words can been explained as a direct result of practicing the same articulations over and over (e.g., Bybee and Hopper, 2001). Neither of these mechanisms, however, predicts paradigmatic enhancement, which seems to require a different explanation.

The first detailed theoretical account of paradigmatic enhancement is given by Cohen (2015), who adopts an exemplar theoretic approach (e.g., Goldinger, 1998) in which the pronunciation of a word is codetermined by all exemplars that are activated during production (e.g., Walsh et al., 2010). According to Cohen (2015), during lexical access, multiple representations of paradigmatically related words may be activated. This coactivation is mediated by the linguistic context, which means that paradigm members that are contextually plausible are activated more strongly. For example, in the Dutch sentence *de antilopen/antilopes rennen* “the antelopes are running,” both the *-en* and the *-s* form are allowed, and, as a result, activation of the *-s* form may lead to coactivation of the *-en* form. Importantly, the degree to which the exemplars of the coactivated form contribute to the pronunciation of the word depends on the number of exemplars of each activated form, i.e., how often the speaker has encountered the respective forms. For instance, the pronunciation of the *-s* suffix in Dutch *antilopes* might be strongly influenced by *antilopen* exemplars because the *-en* form is much more frequent for this noun. Cohen (2015) argues that the nature of this influence can be predicted by comparing the target pronunciation and the coactivated pronunciation. In our example, final [s] in the target pronunciation [ɑntilopəs] would be reduced because the coactivated pronunciation [ɑntilopə] does not have a final [s] (the /n/ in the *-en* suffix is usually omitted). However, if the target form, e.g., *piramides*, is more frequent than the coactivated form, *piramiden*, we would expect the [s] in the target pronunciation to be less reduced. According to this account, then, paradigmatic enhancement reflects a relative lack of reduction due to the relative infrequency of coactivated word forms. While direct phonetic influence of the coactivated variants on pronunciation works for this example and the phenomena described by Cohen (2014) and Cohen (2015), it does not explain other manifestations of paradigmatic enhancement (e.g., Tomaschek et al., 2021b). It may also be that coactivation of paradigmatic alternatives indirectly disrupts articulation of the target form. Bell et al. (2020) propose that enhancement of a particular segment depends on the amount of activation available for its articulation, which in turn is decreased by paradigmatic alternatives with a different (or no) segment in the same position. In such an account, a strong representation of an alternative plural variant would take away activation from the articulation of final [s], resulting in reduced pronunciation. Regardless of the precise implementation of reduction, an account in which articulation is affected by coactivated representations of paradigmatic alternatives may explain why produced forms with higher frequencies relative to paradigmatic alternatives have less reduced pronunciations.

### 1.2. Interactions Between Paradigmatic Relations

While it has been shown that base-complex relations and relations between paradigmatic alternatives affect production, it is unknown whether these different within-paradigm relations interact with each other. Such an interaction might be expected given previous theoretical assumptions about the respective relations. The first assumption is that the base-complex frequency relation (e.g., *piramide-piramides*) reflects the representational strength of complex words (e.g., Hay, 2001). Whether this assumption applies to Dutch variable plurals is tested separately in our Study 1. The second assumption (as proposed by Cohen, 2015) is that the degree of paradigmatic enhancement depends on the representational strengths of the produced form (e.g., *piramides*) and the co-activated alternative form (e.g., *piramiden*). If we apply the definition of representational strength in the first assumption to the second assumption, a hypothesis can be constructed about how paradigmatic enhancement should be affected by an interaction between base-complex relations and relations among paradigmatic alternatives. The first assumption implies that the greatest disparity in representational strength between paradigmatic alternatives can be found if one alternative (A1) is much more frequent than the base form (B) whereas the other alternative (A2) is much less frequent compared to the base (i.e., A2 < B < A1). According to the second assumption, we would expect to see a strong paradigmatic enhancement effect in this case. Conversely, if both alternatives are much less frequent than the base (i.e., A1, A2 < B), we would not expect to see a strong paradigmatic enhancement effect. In terms of processing mechanisms, this means that a paradigmatic enhancement effect would not be expected to surface if production of paradigmatic alternatives might be mostly computational, i.e., if production does not involve strong representations of complex words. Applied to Dutch variable plurals, this interaction hypothesis would predict that the relative frequency of plural variants has a greater effect on pronunciation if the noun paradigm is plural-dominant. After all, in plural-dominant paradigms the differences in representational strength between plural variants are potentially greatest (i.e., A2 < B < A1; or A1 < B < A2). This interaction hypothesis is tested in our Study 2.

Dutch plural variation has a number of features that makes it a suitable phenomenon to test the interaction between base-complex relations and relations among paradigmatic alternatives. Firstly, as the plural variants have the same morphological function (see also *morphological overabundance*; Thornton, 2019), they form paradigmatic alternatives in every context. Consequently, the context of the plural variants does not need to be controlled in an experiment to collect enough data points, which means that the relations among plural variants can be studied in natural communicative settings. Secondly, for Dutch variable plurals the base-complex relation and the relation between paradigmatic alternatives are not conflated. Such a conflation of relations can be found in English verb agreement to collective nouns: in the *the family seem/seems* example, *seem* is both the base form and the alternative of *seems* (see also Cohen, 2014). Finally, the range of relative frequencies between the members of the noun paradigms that contain variable plurals is large enough to measure their effect on pronunciation. Importantly, given the assumption that the frequency of a complex word relative to its base reflects how it is processed, paradigms should be included in which the singular base is more frequent than the complex plurals as well as paradigms with relative frequencies in favor of the plural forms. Conveniently, a fair number of Dutch nouns are plural-dominant, providing the necessary spread in the relative frequency between complex and base forms. In sum, Dutch variable plurals provide an excellent opportunity to investigate how the different relations within paradigms interact during production.

### 1.3. The Present Studies

The current research approaches the interaction between singular-plural relations and relations among plural variants in two studies. The first study tests whether previous assumptions about the singular-plural relation for invariable plurals and base-complex relations in general also apply to variable plurals. The second study of this research tests whether the singular-plural relation interacts with the relation between plural variants in affecting the processing of variable plurals. In both studies, we will focus on how production of a single variant is affected by paradigmatic effects. Specifically, we will focus on the *-s* variant because affixes realized as [s] have reliably shown morphological effects on duration in previous research (e.g., Walsh and Parker, 1983; Cohen, 2014; Plag et al., 2017, 2020; Tomaschek et al., 2021a for *-s* suffix in English; Kuperman et al., 2007 for *-s-* interfix in Dutch).

Our first study tests the association between the base-complex frequency relation and the representational strength of complex words. As strong representations have been argued to limit the influence of generalization (e.g., Divjak and Caldwell-Harris, 2019), this association can be evidenced by showing that relatively frequent complex words are less affected by generalization. Specifically, our first study investigates whether Plural Dominance, measured as the combined frequency of the plural variants divided by the frequency of the singular form, moderates the influence of phonological generalizations on the choice between plural variants. It has been shown that phonological generalizations can be used to accurately predict the plural suffix of many Dutch nouns (Baayen et al., 2002; Keuleers et al., 2007). Given psycho-linguistic studies on Plural Dominance (Baayen et al., 2008; Beyersmann et al., 2015), we would expect that the plural variant of plural-dominant plurals is harder to predict using phonological patterns. In order to test the predictability of a plural variant, we needed a measure of the distribution of plural variants that would be predicted by phonological generalizations and a measure of the actual distribution. The actual distribution can be extracted from a corpus of written Dutch. By counting the number of *-s* (e.g., *piramides*) tokens and the number of competing (e.g., *piramiden*) tokens, the ratio of *-s* tokens, henceforth -s Bias, can be computed for each noun. The predicted distribution can be obtained using a computational model that predicts the plural variant based on phonological features of the noun. We adopted the analogical model of Dutch plural formation described by Keuleers et al. (2007) to predict the plural allomorph of variable plurals. In this model, which is implemented using the TiMBL software (Tilburg Memory Based Learner; Daelemans et al., 2018), conflicts between analogies with different nouns are possible, resulting in uncertainty about the plural allomorph that should be chosen. By expressing this uncertainty as the probability of obtaining the *-s* allomorph and entering it as the -s Prediction variable into a regression model of the -s Bias, we can assess the extent to which phonological generalization predicts the variation. We expect that the positive effect of -s Prediction on the observed -s Bias will be smaller for more frequently pluralized nouns, that is, for nouns with higher Plural Dominance. This outcome would support the hypothesis that the frequency relation between variable plurals and their singular forms reflects the influence of different processing mechanisms (generalization vs. whole-word access) on the production of variable plurals. More generally, such an outcome supports the assumption that the base-complex frequency relation reflects the representational strength of complex words.

In our second study, we test the hypothesis that base-complex relations interact with relations among paradigmatic alternatives. Specifically, we used the paradigmatic enhancement phenomenon to investigate the interaction between the singular-plural dominance relation and the coactivation among plural variants. On the basis of Cohen's (2015) theoretical account of paradigmatic enhancement, we can predict that a plural variant that is infrequent relative to its alternative should be pronounced with a more reduced plural suffix. As such, we expect that final *-s* is shorter for plurals with a more frequent *-en* or irregular variant. Crucially, we expect that this effect of -s Bias is mediated by the Plural Dominance measure. For noun paradigms with high Plural Dominance, a low -s Bias means that the competing plural variant is frequent relative to both the *-s* variant and the singular. As such, the final [s] of these nouns is expected to be shorter due to interference of the much stronger representation of the alternative variant. Conversely, a high -s Bias for plural-dominant nouns suggests that the *-s* variant has a much stronger representation than the alternative variant, which is therefore not expected to reduce the duration of final [s]. For infrequently pluralized nouns, i.e., nouns with low Plural Dominance, we do not expect a strong paradigmatic enhancement effect as neither plural variant is assumed to have a strong representation. These outcomes would provide evidence for an account of plural production in which the representational strength of the plural variants negotiates between the influence of generalizations across different noun paradigms and the influence of alternatives within its own paradigm. In such an account, plural variants that have strong representations are mostly produced by accessing whole word representations, whereas plural variants with weak representations are mostly produced by a generalization mechanism. The influence of the competing plural variant on production is dependent on its representational strength relative to that of the produced variant. More generally, such an outcome would be in line with the hypothesis that base-complex relations interact with relations among paradigmatic alternatives.

## 2. Distributional Study

### 2.1. Materials and Methods

#### 2.1.1. Frequency Data

Most of the variables used in this study (see Table 1) were based on word frequency data. The corpus used to compute these word frequencies had to meet a number of criteria. Most importantly, it needed to be sufficiently large. Numerous examples of variable plurals are discussed in the literature (e.g., de Haas and Trommelen, 1993), but many of these are low frequency words and are therefore not likely to occur frequently in small text corpora, which would hamper the computation of reliable ratios of the occurrence of *-s* vs. other plural affix variants. The second criterion related to the level of annotation. Word tokens needed to be morphologically annotated for the data processing step, which consisted of automatically selecting nouns, identifying which word forms were part of the same inflectional paradigm, and distinguishing between invariable and variable plurals. Finally, we preferred a corpus that was not solely based on formal written language. This was important as formal texts are more sensitive to prescriptive rules and conscious linguistic processing, which might have limited the amount of variation in plural suffixes.

**Table 1:** Mean, minimum and maximum values of the variables in the distributional study.

**Dependent variable**	**Mean**	**Min.**	**Max.**
*-s Bias*	−0.222	−7.749	6.564
**Predictors of interest**			
*-s Prediction*	0.482	0.000	1.000
*Plural Dominance*	−1.079	−7.726	7.953
**Covariate**			
*Plural Frequency*	3.329	1.099	8.033

The SUBTLEX-NL corpus was found to best match these criteria. With more than 400,000 unique, morphologically annotated word forms, it met two of our requirements. Furthermore, it is based on subtitles, which have word frequency distributions that have been shown to predict word processing measures more accurately than frequencies from alternative sources (Keuleers et al., 2010), presumeably because subtitle frequencies approximate those in natural speech. Using the morphological annotations of the SUBTLEX-NL corpus, we automatically separated the nouns that had a single plural form from those that had multiple. As we focused on *-s* plural variants, we only considered nouns with multiple plurals if one of those was an *-s* variant. From this set of variable plurals, we manually excluded nouns that were incorrectly identified as having a variable plural. For instance, certain orthographically identical but phonologically and semantically separate words with different plurals, e.g., *sportster+s* “female athletes” and *sportster+en* “sports stars”, were incorrectly conflated under a single lexical entry. Similarly, we excluded nouns if their different plural forms had separate (though sometimes related) meanings, such as *wortelen* “carots” and *wortels* “roots” (see Haeseryn et al., 1997). Other cases we excluded involved incomplete interfixed compounds, such as *functionerings(gesprek)* “appraisal [meeting]”, which were sometimes analyzed as *-s* plurals by the morphological tagger used for SUBTLEX. Apart from removing obvious mistakes, we also excluded plural forms that occur in very few paradigms such as *brandweerman-brandweerlieden* “firefighter(s)”, and *-en* plurals that could also be analyzed as infinitive verb forms such as *testen* in *De onderzoeker houdt van testen* “The researcher loves tests/to test”. Finally, we removed forms that occurred more frequently in foreign utterances than in Dutch utterances, e.g., *rings*.

After excluding mistakes and potentially unreliable data, the selection of variable plurals consisted of 384 noun types. For each of these nouns the dependent variable -s Bias was computed by dividing the number of *-s* tokens by the number of tokens with the alternative plural variant and taking the natural logarithm of the resulting ratio. Additionally, the predictor Plural Dominance was calculated for each noun type by dividing the total number of plural tokens by the total number of singular tokens and taking the natural logarithm of the resulting ratio. Following Cohen (2015), we expressed these within-paradigm frequency relations using log-transformed ratios to compensate for the enormous range in token frequencies. A positive log-ratio indicates that the numerator (e.g., plural frequency for Plural Dominance) is greater than the denominater (e.g., singular frequency for Plural Dominance). The reverse frequency relation is true for a negative log-ratio, and a log-ratio of zero indicates that numerator and denominator are equally frequent. In other words, -s Bias and Plural Dominance are centered around the point of equal proportion. In addition to the paradigmatic predictors, the Plural Frequency variable was computed by taking the natural logarithm of the total number of plural tokens for each noun. For lower values of Plural Frequency, the -s Bias measure is biased toward 0. In fact, -s Bias is exactly 0 for all variable plurals that occur only twice in the corpus. These plurals were excluded, as they would lead to less reliable estimates of the regression model. The final set consists of 361 noun types. Section 2.1.3 describes how we used Plural Frequency to account for the tendency of *-s Bias* toward 0 in the remaining data when estimating the effects of the predictors of interest.

#### 2.1.2. Generating -s Predictions With TiMBL

In order to model the influence of between-paradigm relations on the choice of plural variant, we needed detailed phonological transcriptions for the nouns that were identified in the SUBTLEX corpus. As such, we used the CELEX corpus (Baayen et al., 1996) to collect phoneme and word stress features for the singulars forms of both the variable and invariable plurals that were selected from SUBTLEX. In addition to these features, we also needed a computational model that could use them to predict the plural variant. We adopted the approach by Keuleers et al. (2007), who used the TiMBL classifier (Daelemans et al., 2018) to implement a probabilistic model based on phonological and orthographic analogy that predicts the suffix of Dutch plurals. In this approach, each plural was represented as a vector of phonological and orthographical features and a class label indicating the correct plural type; see Table 2 for the example *vaders*.

**Table 2:** An example of a TiMBL feature vector and class label for the plural *vaders* “fathers”.

**Penultimate syllable**				**Final syllable**					**Plural type**
**Onset**	**Nucleus**	**Coda**	**Stress**	**Onset**	**Nucleus**	**Coda**	**Stress**	**Final letter**	
v	a	–	+	d	@	r	–	r	-s

In the present study, we recognized 3 plural suffix types: *-s, -en*, and *other*. TiMBL uses the k-nearest neighbors algorithm (kNN) to predict the plural suffix of noun types that are unseen by TiMBL. This algorithm compares the feature vector of an unseen noun to the feature vectors of nouns for which the plurals are known. The noun with the feature vector most similar to that of the unseen noun is the closest neighbor at *k* = 1. Similarly, the second-most similar noun is at distance *k* = 2, *et cetera*. Consequently, if the parameter k is set to larger numbers, more dissimilar nouns are considered in the comparison. In the standard configuration of the kNN algorithm, the unseen noun is assigned the plural type that was associated with the majority of the neighbors. If distance weighting is enabled, closer neighbors count for more than distant neighbors.

Although this standard implementation of TiMBL has been shown to model phonological factors on invariable Dutch plurals quite well (Keuleers et al., 2007), its categorical output is not a very useful predictor for variable plurals. Therefore, we had our TiMBL model produce two types of output: categorical classifications for training and validation based on the invariable plurals, and continuous probabilities for prediction of the variable plurals. Accordingly, we separated our plural data into a *training set*, which consisted of 9908 invariable plural types, a *validation set*, which contained another 1532 invariable plurals, and a *test set*, which contained 361 variable plurals. The model was subsequently trained and optimized on the training and validation sets using categorical labels. This process involved comparing the validation accuracies for every combination of the hyperparameters listed in Supplementary Table 1.

The best validation accuracy of 0.949 was achieved by a model that used inverse distance decay with k=5, trained on type merged data with feature vectors of 2 syllables (see Supplementary Table 1 for descriptions of features). Subsequently, this model was used to provide probabilities of the respective plural classes for the variable nouns in the test set. The predictor of interest -s Prediction (see Table 1) was extracted from the resulting probability distributions.

#### 2.1.3. Modeling -s Bias

To assess the potential for collinearity in our data, we calculated correlations between all the variables in this study. None of the pairwise Pearson correlations between predictor variables exceeded *r* = 0.20 (see Supplementary Figure 1 for full documentation of all correlations).

In choosing an appropriate statistical model of the interaction effect between Plural Dominance and -s Prediction on -s Bias, we considered the nature of the dependent variable. As -s Bias can be described as a log odds ratio, a binomial model seemed the obvious choice. Binomial models are suitable for our data as they can take into account differences in sample size, i.e., plural frequencies, when calculating the standard errors of the estimated log odds. However, when we considered that the dependent variable is based on characteristics of specific words (see *language-as-fixed-effect fallacy*, Clark, 1973), it became clear that regular logistic regression would lead to a poorly estimated model. We know from research on invariable Dutch plurals (Keuleers et al., 2007) that the choice of allomorph does not always follow a predictable pattern. A calculation based on the data from Keuleers et al. (2007) shows that around 9% of invariable plurals does not have the allomorph predicted by TiMBL. In other words, for some nouns the choice of plural allomorph is noun-specific. Likewise, we might expect that the distribution of plural variants for certain variable plurals is at least partly specific to the noun. It is therefore likely that modeling -s Bias using logistic regression would lead to overdispersion, i.e., a case in which the data show more variability than expected on the basis of a regular binomial model. After all, simple logistic regression assumes that the -s Bias of each noun can be predicted exclusively from fixed effects (e.g., phonological patterns). Instead, an approach was needed which treated the underlying probability of an *-s* variant as a random variable. Although random structure in binomial data can be modeled using generalized mixed effects models, previous research has shown that beta-binomial regression more reliably results in robust parameter estimates (Harrison, 2015). Beta-binomial regression assumes that the probability parameter of the binomial model is randomly chosen from a beta-distribution for each noun. The additional free parameter of this beta-distribution is estimated when the beta-binomial model is fitted. This allowed us to model both fixed and noun-specific effects on -s Bias. As such, we used beta-binomial regression, as implemented in the R package aods3 (Lesnoff and Lancelot, 2018), to model -s Bias. Model diagnostics did indeed reveal that a beta-binomial model fitted the data significantly better than a binomial model, see Supplementary Figure 2.

The -s Prediction × Plural Dominance interaction was included to test our hypothesis that the representational strength of a plural limits the degree to which the choice between plural variants is governed by analogical generalization. We expected that higher values of Plural Dominance, which are assumed to reflect stronger plural representations, would be associated with a weaker relation between -s Prediction and -s Bias. Additionally, the -s Prediction × Plural Frequency interaction was included to account for the tendency of -s Bias toward 0 for infrequent plurals. Biased values of -s Bias for low frequency plurals limit the amount of variance that can be explained by -s Prediction. As such, we expected that the positive relation between -s Prediction and -s Bias would diminish for lower values of Plural Frequency. By accounting for this effect, the estimation of the -s Prediction × Plural Dominance interaction should be less influenced by the limited effect of -s Prediction at lower Plural Frequency.

### 2.2. Results

Table 3 summarizes the outcome of the fitted beta-binomial model of -s Bias. The μ coefficients describe the average relations between the predictors and -s Bias. The ϕ coefficient, a dispersion parameter, describes the estimated shape of the underlying probability distribution of -s Bias.

**Table 3:** Beta-binomial model of -s Bias.

**μ Coefficients**	**Estimate**	**Std. Error**	***z*** **-value**	***p***
*Intercept*	0.588	0.277	2.123	0.034
*-s Prediction*	−1.238	0.497	−2.493	0.013
*Plural frequency*	−0.379	0.063	−5.974	0.000
*Plural dominance*	−0.058	0.069	−0.833	0.405
*-s Prediction* : *Plural frequency*	0.614	0.114	5.392	0.000
*-s Prediction* : *Plural dominance*	−0.248	0.120	−2.069	0.039
**ϕ Coefficients**	**Estimate**	**Std. Error**		
*Intercept*	0.350	0.016		

*p-values were estimated using Wald tests*.

**Figure 1:**
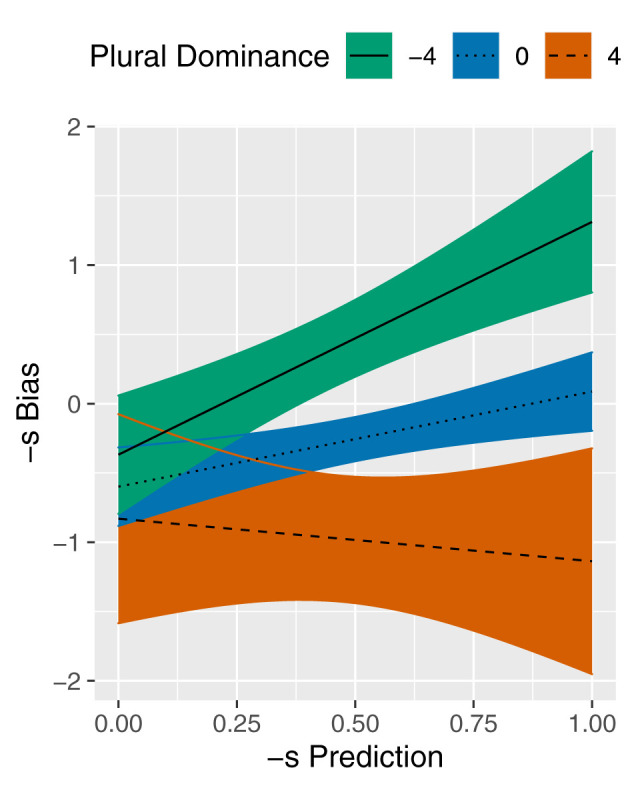
Partial effect plot for the -s Prediction × Plural Dominance interaction in the model of -s Bias. Plural Frequency is held constant at the median value. Coloured bands reflect 95% confidence intervals.

Table 3 reveals a significant interaction between the predictors of interest, -s Prediction and Plural Dominance. The fitted lines in Figure 1 illustrate the estimated effect of -s Prediction on -s Bias at different values of Plural Dominance. A Plural Dominance of 4 amounts to a plural/singular ratio of more than 50/1 and it is indicated by the dashed line with an orange confidence band; a value of 0 corresponds to a plural/singular ratio of exactly 1/1 which is represented by the dotted line with a blue confidence band; and a value of –4 reflects a plural/singular ratio of less than 1/50 and it is visualized by the solid line with a teal confidence band. As Plural Dominance decreases, the slopes of these lines increase. This result is in line with our expectations, which suggested that generalization, represented by -s Prediction, mainly affects the plural variation of plurals with less representational strength (Plural Dominance).

**Figure 2:**
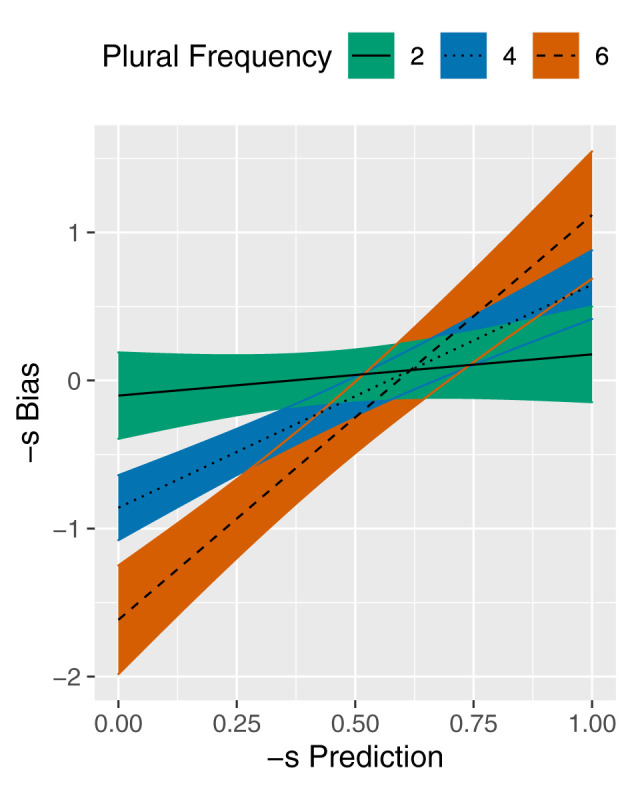
Partial effect plot for the -s Prediction × Plural Frequency interaction in the model of -s Bias. Plural Dominance is held constant at the median value. Coloured bands reflect 95% confidence intervals.

Additionally, Table 3 indicates a significant interaction between -s Prediction and Plural Frequency. The fitted lines in Figure 2 visualize the effect of -s Prediction on -s Bias at different values of Plural Frequency. The log-transformed values of 2, 4, and 6 correspond to approximate untransformed frequencies of 7, 55, and 403, respectively. As illustrated by the nearly horizontal line, -s Prediction does not have a clear effect on -s Bias for nouns with low Plural frequency. Conversely, for nouns with high Plural frequency, the rising line indicates a positive effect of -s Prediction on -s Bias. This interaction was expected because -s Bias has a tendency toward 0 for low frequency nouns.

## 3. Durational Study

### 3.1. Materials and Methods

#### 3.1.1. Acoustic Data

The speech material analyzed in this study was extracted from the Dutch speech corpora listed in Table 4. We limited our dataset to Netherlandic Dutch, as the Dutch-Belgian border coincides with a different distribution of plural allomorphs for a number of nouns Goeman et al. (2005). Variable plural tokens were automatically identified using the orthographic transcriptions of the speech corpora and the selection of 361 noun types that occurred with multiple plural forms in SUBTLEX. We arived at a final dataset after discarding observations that would have resulted in unreliable duration measurements. This included tokens in which the final /s/ was preceded or followed by /s/, /z/, /ʃ/, /ʒ/, /t/, /d/ or /j/, as it is very difficult to segment the speech signal into two distinctive sounds in such cases. Furthermore, in certain recordings that involved multiple speakers, the respective speakers' voices were not recorded on separate audio channels. As a result, overlapping speech in those recordings is more difficult to segment, and durational measurements of such data may not be reliable. Therefore, final /s/ tokens from these recordings were excluded if they occurred in overlapped speech. The final data set consisted of 594 *-s* plural tokens.

**Table 4:** Overview of the corpora used in the durational study, including the number of *-s* plural tokens that were selected.

**Register**	**Corpus**	**Reference**	**Tokens**
Spontaneous conversation	Spoken Dutch corpus part a	Oostdijk, 2000	196
	Spoken Dutch corpus part c		47
	Spoken Dutch corpus part d		45
	Ernestus corpus of spoken Dutch	Ernestus, 2000	9
	IFA dialog video corpus	van Son et al., 2008	8
News broadcasts	Spoken Dutch corpus part k	Oostdijk, 2000	74
Read stories	Spoken Dutch corpus part o		215
		**Total:**	594

The final /s/ duration of the variable plural tokens was measured by the Kaldi-based (Povey et al., 2011) CLST forced-aligner (Kuijpers et al., 2018) to limit the influence of human biases and inconsistencies. The pronunciation dictionary of the forced-aligner was enriched to allow for reduced pronunciation variants according to the rules laid out by Schuppler et al. (2011). The parameters of the forced-aligner were validated on a separate set of manually annotated utterances in the Spoken Dutch Corpus (Oostdijk, 2000). Using this procedure we selected the settings that resulted in the smallest number of phonetic feature changes, insertions or deletions (as measured by *weighted feature edit distance*; Mortensen et al., 2016) between the automatic and manual transcriptions. The extracted segment durations from the automatically aligned speech were log-transformed to arrive at our dependent variable -s Duration.

#### 3.1.2. Predictors

Our paradigmatic predictors of interest -s Bias and Plural Dominance were extracted from the data set used in the distributional study. Additionally, we used SUBTLEX to calculate two alternative measures of lexical representation, -s Frequency and Relative -s Frequency, which have been used in previous research. -s Frequency was computed to represent an account of the lexical representation of the *-s* plural based on its log-transformed absolute frequency instead of paradigmatic relations (e.g., Schuppler et al., 2012). We also included the Relative -s Frequency to account for the proposal that paradigmatic effects should be measured by dividing the frequency of the *-s* plural by the lexeme frequency and log-transforming the resulting proportion (e.g., Cohen, 2015).

In order to account for the variance in -s Duration that is unrelated to our paradigmatic predictors, we included a number of covariates. Specifically, we used covariates that have been used in previous studies that looked at segmental durations in corpus data (e.g., Plag et al., 2017).

One of the more obvious influences on segmental duration comes from the relative speed with which the surrounding speech is uttered. We measured this influence using two different variables. Firstly, Speech rate was calculated in syllables per second by counting the number of syllables in the current utterance and dividing it by the duration of the utterance. Utterances were defined as uninterrupted chunks of speech. The number of syllables was determined by counting the number of vowels that were recognized by the forced aligner. Secondly, Base duration was defined as the natural logarithm of the duration of the word excluding the final /s/. This measure was included to account for the variation in local speaking rate that was not captured by the speech rate variable.

The duration of final /s/ might also be influenced by the phonological characteristics of the word containing and the word following it. As such, Number of syllables was included as a variable to account for the segmental reduction that increases with the number of syllables in a word (e.g., Nooteboom, 1972). Additionally, the phonetic class of the Previous segment was taken into account, as it might influence the duration of the final /s/. For instance, final /s/ might be shorter if it forms a consonant cluster with the preceding segment (e.g., Klatt, 1976). The phonetic context following final consonants has also been shown to influence segmental duration (e.g., Luce and Charles-Luce, 1985). Therefore, the broad phonetic class of the Next segment was also included as a variable. We considered the following classes for Previous segment and Next segment: *vowels, liquids, approximants, nasals, fricatives, plosives* and *silence*.

A number of prosodic variables have been shown to affect the pronunciation of consonants (e.g., Cho and McQueen, 2005). On a word level, stressed syllables result in longer segments. Therefore, we used CELEX to implement Word stress as a categorical variable which indicated whether the stressed syllable contained the final /s/. The larger prosodic context also influences segmental duration (Cho and McQueen, 2005). Particularly relevant for the current study is the phenomenon known as final lengthening, in which segments that occur before a prosodic boundary are lengthened (e.g., Hofhuis et al., 1995). Unfortunately, the corpora used in this study were not prosodically annotated. To get around this problem some corpus studies (e.g., Plag et al., 2017) use syntactic boundaries instead, as these sometimes co-occur with prosodic boundaries. We took a similar approach by generating syntactic annotations using the dependency parser (Canisius et al., 2006) included in the FROG natural language processing tool (Hendrickx et al., 2016). We then derived features from these annotations that have been shown to predict prosodic boundaries, such as intermediate or intonational phrase breaks (see features F2–F8 in Ingulfsen, 2004, pp. 36–38). In order to limit the number of prosodic boundary variables, we used a principle component analysis to identify 5 principle components, Prosody_*PC*1−5_, that accounted for more than 94% of the variance described by the 7 original features.

We also considered the distributional characteristics of the words containing and surrounding the /s/. It has been shown, for instance, that words which are predictable given the surrounding words have more reduced realizations (e.g., Pluymaekers et al., 2005; Bell et al., 2009). As such, we used the NLCOW14 corpus (Schäfer, 2015) to measure the bigram frequency of the plural and the word preceding it in addition to the bigram frequency of the plural and the word following it. By dividing these respective bigram frequencies by the frequency of the plural form in the NLCOW14 corpus, we calculated conditional probabilities of the plural form given the preceding and subsequent word. These were log-transformed, resulting in Probability from previous word and Probability from next word, respectively. Similarly, whether or not a word has been recently mentioned may also affect its pronunciation (e.g., Pluymaekers et al., 2005). This was encoded as the binary Recently mentioned variable by checking whether the same plural had been uttered in the 30 seconds prior.

Another feature that may influence phonetic reduction concerns a word's phonological similarity to other words. This similarity has been implemented by counting the number of Phonological neighbors, which are the words that differ from the target word by one sound. Higher neighborhood density has been associated with both more and less reduced segments (see discussion in Gahl et al., 2012). For each plural, we used the pronunciation lexicon that came with the CLST forced aligner (Kuijpers et al., 2018) to find the number of lexical neighbors.

Finally, previous research has shown that more careful speech is associated with longer durations (e.g., van Son and Pols, 1999). We expected that some of the speech used in this study, such as the read-aloud stories, would be more careful compared to speech from spontaneous conversations. Consequently, speech Register was the final influence on the duration of final /s/ that we considered. This variable had three levels: *Conversation, Stories* and *News*.

#### 3.1.3. Modeling -s Duration

We used linear mixed effects regression, as implemented in the R package lme4 (Bates et al., 2015), to model -s Duration. By analyzing the effect of the interaction between -s Bias and Plural Dominance on -s Duration we hoped to test our hypothesis that the effect of competition between plural variants on pronunciation is more noticeable if the plural variants are representationally strong relative to the singular. Additionally, we wanted to know how well our paradigmatic predictors explained differences in -s Duration compared to alternative measures like the absolute -s Frequency. As such, we created multiple models.

First, we fitted a *Paradigmatic model* containing -s Bias, Plural Dominance and their interaction term, all covariates, and random intercepts for Speaker and Noun, which was the maximal random structure that was supported by the data. Additionally, we fitted two alternative models in which the -s Bias and Plural Dominance variables were replaced by alternative measures of representational strength. In the *Absolute frequency model* we replaced the paradigmatic measures with a single -s Frequency predictor. We also fitted a *Relative frequency model*, in which we used the Relative -s Frequency measure. Using the AIC scores of the resulting three models, we calculated their relative likelihood to determine whether our paradigmatic predictors provided the best fit to the data.

Subsequently, we wanted to interpret the predictors of interest in our paradigmatic model. As such, we needed to avoid collinearity between our predictors of interest and any covariates. To assess the potential for collinearity in our data, we calculated correlations between all covariates and our predictors of interest; see Supplementary Figure 3. This showed us that -s Bias was correlated (Pearson's *r* ≥ 0.4) with the covariates Word stress and Number of syllables. This was not very surprising, as both of these covariates can be related to the stress pattern of a noun, which has been shown to affect the choice of plural suffix (Baayen et al., 2002). Removing these covariates would make sure that they could not lead to collinearity issues. However, we wanted to make sure that any potential effect of -s Bias and its interaction with Plural Dominance would not actually be better modeled by the correlated covariates. Therefore, we fitted three linear regression models of -s Duration: for -s Bias, Word stress and Number of syllables, respectively. Each model contained one of the three correlated variables, the Plural Dominance variable and their interaction. An AIC comparison showed that the model containing -s Bias performed best. As such we excluded the correlated covariates from further analysis. Starting from the resulting *Paradigmatic model*, we used backward elimination (as implemented in Kuznetsova et al., 2017) on to arrive at a model in which only the significant predictors remained. After fitting the model with the remaining variables, we trimmed the data with residuals that exceeded 2.5 standard deviations and refitted the model on the trimmed data set, following Baayen (2008). The residuals of this final model were approximately normally distributed, see Supplementary Figure 4.

### 3.2. Results

The full paradigmatic model of -s Duration containing the -s Bias × Plural Dominance interaction had an AIC of 690.90. By comparison, the best performing alternative model, which contained the -s Frequency predictor, had an AIC of 697.86; see Supplementary Table 2 for full models. This means that the *Absolute frequency model* was $exp\left(\frac{690.90-697.86}{2}\right)=0.031$ times as likely to minimize the information loss compared to the *Paradigmatic model* (Burnham and Anderson, 2004). In other words, the *Paradigmatic model* performed much better than the models with alternative measures of representational strength.

**Figure 3:**
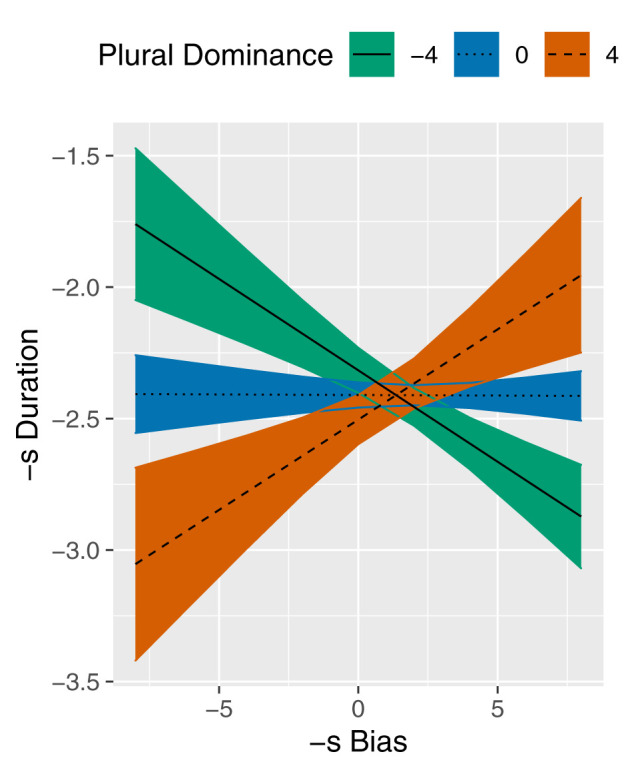
Partial effect plot for the -s Bias × Plural Dominance interaction in the model of -s Duration. Coloured bands reflect 95% confidence intervals.

Table 5 summarizes the parameters of the final model, that is, the *Paradigmatic model* after removal of correlated covariates and insignificant predictors. In addition to the -s Bias × Plural Dominance interaction, this model contains the covariates Speech rate, Prosody_*PC*2_, Next Segment, and Register and the random variable Speaker. As indicated by the estimates in Table 5, the covariates show the expected effects, e.g., a higher Speech rate reduces -s Duration and a subsequent *Silence* is associated with a longer -s Duration. Importantly, Table 5 also reveals a significant interaction between the predictors of interest, -s Bias and Plural Dominance. The fitted lines in Figure 3 illustrate the estimated effect of -s Bias on -s Duration at three different values of Plural Dominance (see section 2.2 for interpretation of these values). At high Plural Dominance, the slope of the line is positive, which means that final *-s* becomes longer if -s Bias becomes larger. This result supports the expected *paradigmatic enhancement* effect. Unexpectedly, we find the opposite effect at low Plural Dominance: for these nouns, final *-s* becomes shorter as -s Bias becomes larger. We expected that -s Bias would have very little effect on -s Duration at negative Plural Dominance, resulting in a horizontal line. However, the model predicts that the *paradigmatic enhancement* effect is already nullified at a Plural Dominance of zero. In noun paradigms with negative Plural Dominance, a reduction effect is predicted.

**Table 5:** Mixed effects model of -s Duration.

**Fixed effects**	**Estimates**	**Std. Error**	***t*** **-value**	***p***
*Intercept*	−2.610	0.038	−68.979	0.000
*Speech rate*	−0.142	0.018	−7.891	0.000
*Prosody*_*PC*2_	0.034	0.016	2.124	0.034
*Next segment: Approximant*	−0.334	0.071	−4.713	0.000
*Next segment: Fricative*	−0.187	0.047	−3.982	0.000
*Next segment: Liquid*	−0.219	0.191	−1.145	0.253
*Next segment: Nasal*	−0.077	0.078	−0.995	0.320
*Next segment: Plosive*	−0.096	0.070	−1.369	0.172
*Next segment: Silence*	0.562	0.042	13.523	0.000
*Register: Stories*	0.170	0.039	4.374	0.000
*Register: News*	0.016	0.069	0.233	0.817
*-s Bias*	−0.000	0.007	−0.058	0.954
*Plural Dominance*	−0.023	0.010	−2.432	0.015
*-s Bias* : *Plural Dominance*	0.017	0.004	4.271	0.000
**Random effects**	**Variance**	**Std. Deviation**		
*Speaker (Intercept)*	0.019	0.137		
*Residual*	0.125	0.353		

*p-values were calculated using Satterthwaite's method. Reference levels are* Next segment: *Vowel and* Register: *Conversation*.

## 4. Discussion

The current research explored how paradigmatic structure relates to the mechanisms that are involved in the processing of complex words. Dutch variable plurals were chosen as the subject of inquiry, as they are involved in paradigmatic relations that have been associated with generalization, storage and coactivation mechanisms.

In our first study we investigated whether the singular-plural frequency relation of a noun influences the distribution of its plural variants in a Dutch subtitles corpus. We hypothesized that the distribution of variants for nouns with higher Plural Dominance would be less predictable by a measure of phonological generalization. The results supported this account by showing that the positive effect of the generalization measure -s Prediction on the distributional measure -s Bias decreases with higher values of Plural Dominance. These findings are in line with previous accounts of *in*variable plurals (Baayen et al., 2008; Beyersmann et al., 2015) which suggest that higher plural dominance limits the influence of generalization on plural processing. Presumably, plural-dominant variable plurals are less affected by generalization because they have representations that are more stable or are easier to retrieve during the speech production process. The distributional results also contribute to the wider discussion about the role of token frequency in the generalization of morphological exponents. Whereas, previous distributional research has generally focused on absolute token frequency as an inhibitor of generalization (e.g., Cuskley et al., 2014), this study showed that frequency relative to the base form may also affect the scope of general phonological patterns^1^ (see also Tiersma, 1982; Collie, 2008).

The goal of the second study was two-fold. Firstly, we wanted to investigate whether the *paradigmatic enhancement hypothesis* applies to Dutch variable plurals. That is, an *-s* plural variant that is more frequent than its alternative should be phonetically enhanced compared to an *-s* variant that is less frequent than its alternative. This hypothesis was based on the assumption that the more frequent *-s* variant has a stronger representation and therefore its pronunciation is less affected by the coactivated representation of the alternative variant. Additionally, we hypothesized that such a paradigmatic enhancement effect should primarily occur in noun paradigms with relatively high Plural Dominance. This qualification was based on the assumption that the representational strengths of plural variants primarily depend on their frequencies relative to the singular. As such, we expected that the differences in representational strengths measured by -s Bias would be greatest at high Plural Dominance. The results revealed an interaction effect of -s Bias and Plural Dominance on -s Duration. For plural-dominant plurals, a higher -s Bias was associated with a longer -s Duration, which suggests that paradigmatic enhancement is reflected in our data. This finding supports previous accounts of paradigmatic enhancement that interpret the frequency relation between paradigmatic alternatives as a measure of their relative representational strengths Cohen (2014, 2015). Furthermore, the results showed that an increased -s Bias was associated with a shorter -s Duration for singular-dominant plurals, which was surprising as we expected that the pronunciation of infrequently pluralized plurals would not be affected by the frequency relation between variants. Nonetheless, this interaction effect was in line with our hypothesis that paradigmatic enhancement would primarily affect plural-dominant plurals. As such, this study is the first to provide evidence that, in certain paradigms, paradigmatic enhancement is mediated by the base-complex relation.

By combining the findings from both studies, we might better understand the unexpected reduction effect of -s Bias on -s Duration for singular-dominant plurals that was observed in our second study. The interpretation of the -s Bias predictor is crucial to this understanding. The combined results suggest that what -s Bias represents depends on the value of Plural Dominance. At high Plural Dominance, the paradigmatic enhancement effect in the durational study suggests that -s Bias is a measure of the representational strength of the *-s* variant relative to its competitor. However, at low Plural Dominance, the distributional study suggests that -s Bias represents the amount of phonological support from similar paradigms, i.e., the -s Prediction. In formulating the hypotheses for the duration study, we did not consider that increased analogical support could result in the reduction of final *-s*, given the lack of precedents for such an effect (but see *gang size* effect in Tucker et al., 2019). However, the association of reduced final *-s* with increased -s Prediction would fit the more general theory that predictable linguistic elements are reduced (e.g., Bell et al., 2009). Importantly, as this explanation assumes that -s Bias primarily reflects -s Prediction for singular-dominant nouns, it does not conflict with our account of paradigmatic enhancement, which mostly affects frequently pluralized nouns.

The combined results have implications for psycholinguistic models of morphological processing. These models can be categorized according to the relative importance they attribute to abstract rules and lexical storage (see the overviews in Arndt-Lappe and Ernestus, 2020; Fábregas and Penke, 2020). At one end of the spectrum are models that emphasize the role of rules in explaining the paradigmatic structure that arises from commonalities in form and function among the words of a language. In these models, complex words are only stored if they do not submit to morphological rules (e.g., Wunderlich, 1996). Such models often assume that stored exceptions to the rule do not influence regular application of the rule. Our results suggest that the base-complex frequency relation indicates the extent to which variable plurals follow the morpho-phonological rules. As this frequency relation must be stored somehow, either in representations of individual nouns or in weighted connections between morphological exponents and specific semantic representations, the current research questions the complete separation of generalization and storage in rule-based models.

In a second category of models, both abstract rules and lexical storage may affect the production of complex words (see Arndt-Lappe and Ernestus, 2020). In one such a model, the *Parallel Dual Route Model* (e.g., Baayen et al., 1997b), production of a complex word involves simultaneous retrieval of the complex representation and composition involving the base representation. The relative speed of the composition and retrieval routes determines which route affects production the most. In some dual route models, complex words that are less frequent than their bases are assumed to be more easily (de)composable Hay (2001), which would speed up the (de)composition route. This conceptualization of the base-complex relation can be applied to the Plural Dominance variable in our studies. It would predict that singular-dominant plurals are produced using the composition route, whereas plural-dominant plurals are primarily produced using the retrieval route. Such an account would explain why the distribution of plural variants in singular-dominant noun paradigms follows phonological patterns. It would also explain why plural variants in plural-dominant noun paradigms are subject to paradigmatic enhancement. At high Plural Dominance, an *-s* variant with high -s Bias would be more frequent than the singular. As such it would be produced using the retrieval route, and its pronunciation would not be affected by the alternative variant. However, an *-s* variant with low -s Bias would likely be less frequent than the singular while its alternative would be more frequent compared to the singular. The *-s* variant would then be produced using the composition route, and its pronunciation would be affected by the alternative variant, which was simultaneously activated through the retrieval route.

In a third category of models, processing of complex words involves no abstract computation. In those word-based models (e.g., Bybee, 1995), paradigmatic enhancement findings are easily accounted for, as all word forms including their frequencies of occurrence can be stored. Analogy between stored word forms can be used to explain morpho-phonological patterns across paradigms. Our first study showed that such an analogical mechanism can also account for variation observed for Dutch variable plurals. The reduced influence of analogy on the production of plural-dominant nouns can be explained through a weaker activation level of the singular representation relative to the plural representation: a relatively infrequent singular form results in decreased activation of a noun's singular representation, which, in turn, leads to decreased analogical influence of other noun paradigms with phonologically similar singular forms. As such, models without a separate rule-based processing route can account for the Dutch variable plural data as well.

The current findings shed light on how paradigmatic relations may be related to the mechanisms that are involved in the processing of complex words. While the results cannot be explained by the mechanisms of a primarily rule-based model, both a dual-route model and a word-based model are compatible with the results. Regardless of theoretical framework, the novel implication of this research is that the role of the base-complex relation, whether it is conceptualized using activation levels or (de)composability, should be considered when the effect of additional within-paradigm relations, such as those between plural variants, are investigated. It follows that measures which conflate base-complex relations and relations among paradigmatic alternatives, such as form frequency relative to lexeme frequency, might not adequately capture how processing mechanisms interact. This was evidenced in our durational study by the fact that the model which distinguished between -s Bias and Plural Dominance predictors performed much better than the model that combined them into a single Relative -s Frequency predictor. More generally, these findings show that the nature of the individual morphological relations within a paradigm should be considered when their effect on processing is investigated.

In addition to providing answers about paradigmatic relations, our findings also raise questions. This research was concerned with paradigmatic relations and their psycholinguistic relevance during speech production. It would therefore be interesting to know whether our interpretations of the -s Bias, Plural Dominance and -s Prediction relations are representative for the processing mechanisms of individual speakers. However, these relations were measured using type and token frequencies from corpus data. As Blumenthal-Dramé (2012) points out, corpus frequencies do not necessarily reflect the input frequencies of individual language users, but rather a simplified and likely biased approximation of the input of multiple language users. With regard to Dutch plurals in particular, it seems unlikely that all speakers encounter and/or produce the different variants of a plural with the same -s Bias. Presumably, this also leads to differences among speakers in the processing of variable plurals. It is therefore likely that the paradigmatic effects found in this research do not affect the speech of all language users equally. This is particularly true for the distributional study, as it does not relate the paradigmatic measures to the production of individual speakers. Unfortunately, the small size of our data set meant that we could not investigate inter-speaker differences in the paradigmatic enhancement effect. Additionally, due to the nature of the data, we could not take other potentially relevant factors, such as register, into account in our distributional study. These issues may be addressed by studies with better control over the relevant variables.

Furthermore, the findings from the durational study are primarily relevant for a narrow definition of paradigmatic enhancement. In this account, the coactivation resulting in paradigmatic enhancement only involves paradigm members that occur in the same linguistic context. In other words, the context works as a filter that determines which representations are coactivated: in utterances like *the boy runs/run/running* only one paradigm member (*runs*) is likely and therefore no paradigmatic enhancement effect would be expected. As such, this account does not provide clear explanations of paradigmatic enhancement effects on forms that can be predicted from the communicative context (e.g., Kuperman et al., 2007; Schuppler et al., 2012). Our research does provide naturalistic support for previous experimental findings of paradigmatic enhancement in which the linguistic context was controlled to allow for multiple paradigm members (e.g., Cohen, 2014, 2015; Bell et al., 2020; Tomaschek et al., 2021b).

Regardless of the limitations of the current research, its relevance is not limited to obscure morphological alternations. As documented by work on morphological overabundance (e.g., Thornton, 2019), the existence of paradigmatic alternatives is far from exceptional. As such, this research paves the way for similar investigations of paradigmatic relations using other overabundance phenomena. Apart from highlighting the underexplored variation in the realization of complex words, such research would contribute to morphological theory by identifying paradigmatic effects on processing that must be accounted for by psycho-linguistic models. As this research has emphasized, those paradigmatic effects can only be understood if paradigmatic relations are considered both individually and taken together.

## Data Availability Statement

The original contributions presented in the study are publicly available. This data can be found here: https://doi.org/10.17026/dans-xvr-qscf.

## Ethics Statement

The studies involving human participants were reviewed and approved by Ethics Assessment Committee Humanities Radboud University. The patients/participants provided their written informed consent to participate in this study.

## Author Contributions

TZ conducted the research and wrote the initial draft of the manuscript. LB, ME, and IP provided feedback and suggestions for the research, and contributed to the writing of the manuscript. All authors contributed to the article and approved the submitted version.

## Conflict of Interest

The authors declare that the research was conducted in the absence of any commercial or financial relationships that could be construed as a potential conflict of interest.

## Publisher's Note

All claims expressed in this article are solely those of the authors and do not necessarily represent those of their affiliated organizations, or those of the publisher, the editors and the reviewers. Any product that may be evaluated in this article, or claim that may be made by its manufacturer, is not guaranteed or endorsed by the publisher.

## Footnotes

^1^A reviewer pointed out that the interaction effect of our absolute token frequency measure, Plural Frequency, and our generalization measure, -s Prediction, seems to suggest that higher token frequency facilitates generalization, which would be completely contrary to previous findings. However, this interaction is confounded by sampling bias: a frequent plural is more likely to show variation compared to an infrequent plural, especially when the general patterns strongly favour a particular variant. As we only included plurals that showed variation in our data set, this resulted in a better match between -s Prediction and observed -s Bias for high frequency plurals. The interaction between the absolute token frequency and the generalization measure was included to account for the effect of this bias in the estimation of the other predictors, see section 2.1.3.
