# Supplementary material for: Paradigmatic Relations Interact During the Production of Complex Words: Evidence From Variable Plurals in Dutch
Source: Front Psychol. 2021 Sep 1;12:720017. doi: 10.3389/fpsyg.2021.720017 (PMC8442732; doi:10.3389/fpsyg.2021.720017)
Supplement: Supplementary file 1 [file Data_Sheet_1.PDF]

Supplementary Material

APPENDIX A. GRID SEARCH RESULTS ON TIMBL PARAMETERS

Table S1. A list of all hyperparameter names, a short description, and the mean accuracy achieved in the grid search of the TiMBL model.

Parameter	Description	Values	Mean Accuracy	SD
Type Merging	Removes duplicate feature vectors from training data.	No	0.938	0.008
		Yes	0.936	0.011
Number of Syllables	Determines the number of word-final syllables for which phonological features are included.	1	0.926	0.014
		2	0.940	0.006
		3	0.941	0.003
		4	0.940	0.002
k	Determines the maximum distance at which nouns are still considered in the plural prediction.	1	0.939	0.006
		2	0.937	0.007
		3	0.937	0.008
		4	0.936	0.013
		5	0.934	0.014
Distance Weighting	Determines whether distant nouns are less (Inverse Distance Decay) or equally (Zero Decay) important in plural prediction.	Zero Decay	0.933	0.012
		Inverse Decay	0.940	0.005

The hyperparameter space was limited based on the grid search results of Keuleers and Daelemans (2007). As a consequence, a limited number of k values were considered, the onset-nucleus-coda feature scheme was used, features were weighted using the gain ratio method, and similarity was determined using the overlap metric.

APPENDIX B. ADDITIONAL FIGURES FROM DISTRIBUTION STUDY

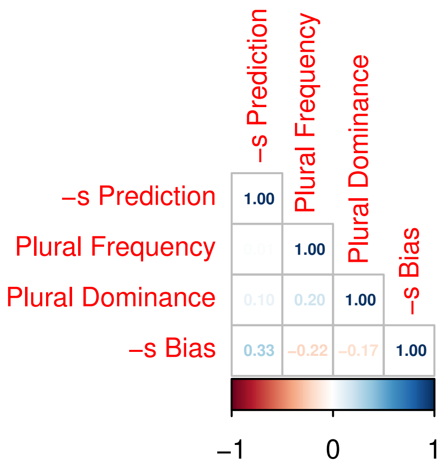

Figure S1. Correlations (Pearson's *r*) between variables in distribution study.

As Figure S2 shows, most of the residuals in the Beta-binomial model fall within the simulated envelope, which suggests a good fit to the data (de Andrade Moral et al., 2017). A Likelihood Ratio Test shows that the beta-binomial model fits significantly better than the binomial model, $\chi(1) = 17349.24$, $p = .000$.

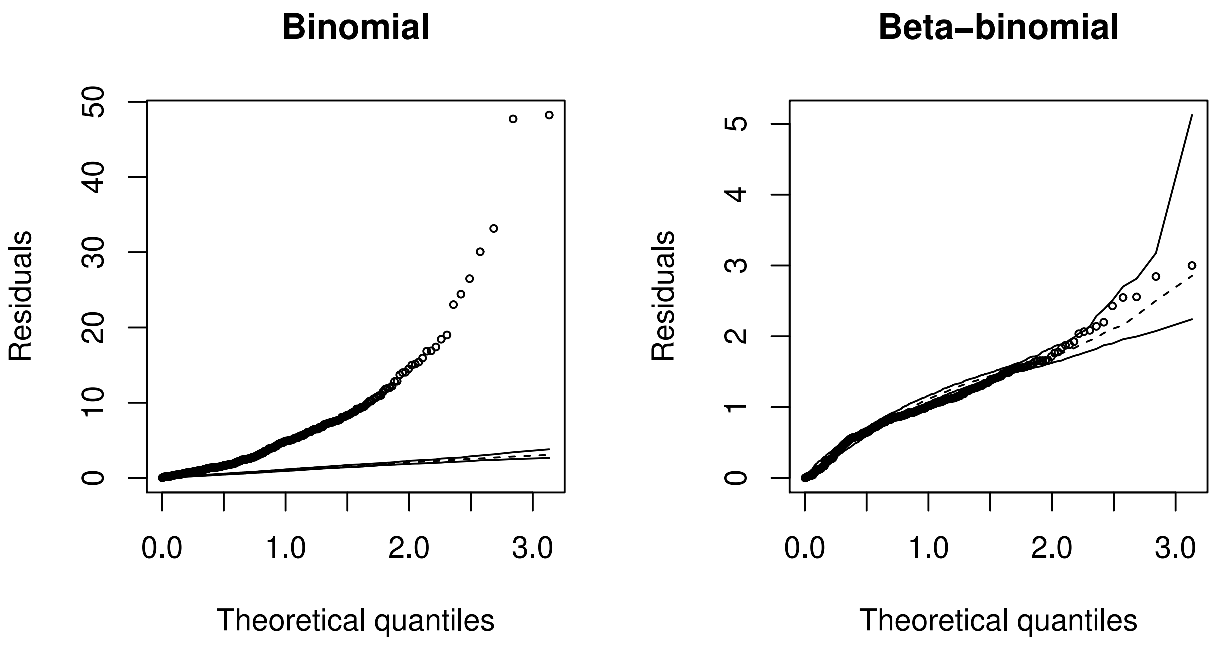

Figure S2. Half-normal plots for binomial and beta-binomial models of -S BIAS. The solid lines represent the 2.5 and 97.5 percentile of simulated residual values.

APPENDIX C. ADDITIONAL FIGURES FROM DURATION STUDY

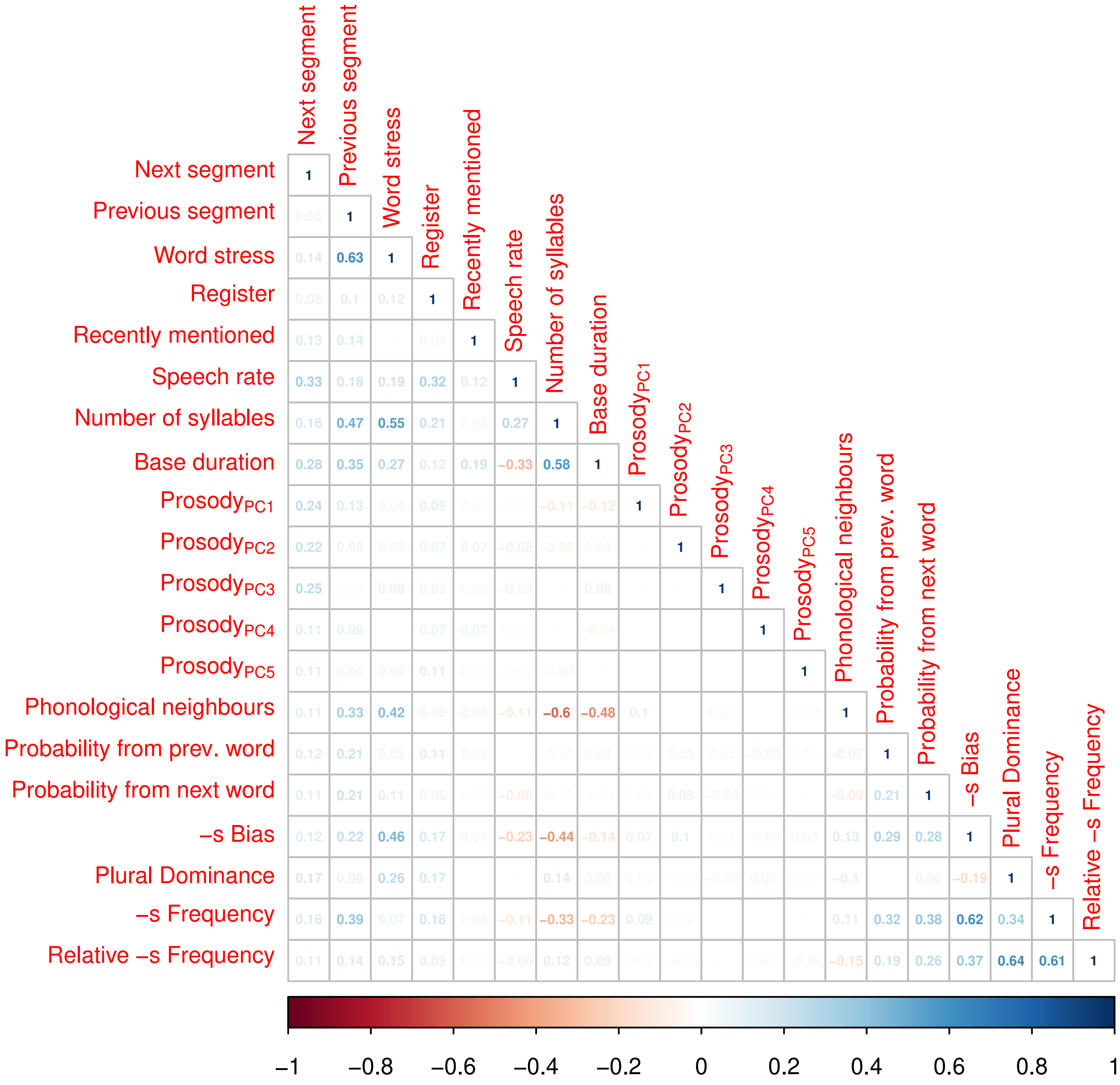

Figure S3. Associations between variables in duration study. Pearson's r was calculated for association between a continuous variable and a continuous or categorical variable. Bias-corrected Cramér's V was calculated for associations between two categorical variables.

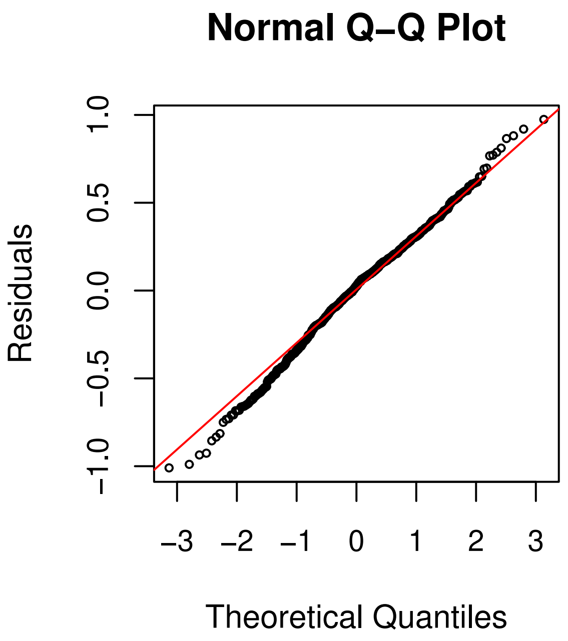

Figure S4. Quantiles of the residuals of the -S DURATION model plotted against theoretical quantiles of a normal distribution.

APPENDIX D. FULL MODELS FROM DURATION STUDY

Table S2. Full mixed effects models of -S DURATION. Numbers in bold represent significant effects using Satterthwaite's method. Significance levels are indicated by *** ($p < .001$), ** ($p < .01$), * ($p < .05$), and . ($p < .1$). Reference levels are NEXT SEGMENT: *Vowel*, PREVIOUS SEGMENT: *Vowel*, WORD STRESS: *Unstressed*, PREVIOUSLY MENTIONED: *False*, and REGISTER: *Conversation*. We arrived at the parameter estimates and standard errors after fitting the respective models on the full data set, excluding data for which the residuals exceeded 2.5 standard deviations and refitting the respective models on the respective trimmed data sets. AIC values were based on models that were fitted on the full dataset using Maximum Likelihood estimation instead of Restricted Maximum Likelihood estimation, which was used for the reported parameter estimates. Marginal and Conditional R^2 were estimated using the MuMIn package (Bartoń, 2020).

	Paradigmatic model		Absolute frequency model		Relative frequency model	
Fixed Effects	Estimate	Standard Error	Estimate	Standard Error	Estimate	Standard Error
Intercept	**-2.563**	**0.045** ***	**-2.482**	**0.078** ***	**-2.617**	**0.057** ***
Speech rate	**-0.132**	**0.022** ***	**-0.132**	**0.022** ***	**-0.133**	**0.022** ***
Base duration	0.026	0.027	0.027	0.028	0.030	0.028
Prosody$_{PC1}$	**-0.037**	**0.016** *	**-0.039**	**0.016** *	**-0.038**	**0.017** *
Prosody$_{PC2}$	0.032	0.016 .	0.028	0.016 .	0.027	0.016 .
Prosody$_{PC3}$	0.028	0.016 .	0.028	0.016 .	0.027	0.016 .
Prosody$_{PC4}$	**-0.033**	**0.016** *	**-0.036**	**0.016** *	**-0.036**	**0.016** *
Prosody$_{PC5}$	0.004	0.016	0.005	0.016	0.005	0.016
Next segment: Approximant	**-0.355**	**0.073** ***	**-0.350**	**0.073** ***	**-0.349**	**0.073** ***
Next segment: Fricative	**-0.183**	**0.048** ***	**-0.183**	**0.049** ***	**-0.179**	**0.049** ***
Next segment: Liquid	-0.292	0.194	-0.325	0.194 .	-0.308	0.194
Next segment: Nasal	-0.098	0.080	-0.107	0.080	-0.109	0.080
Next segment: Plosive	-0.053	0.072	-0.050	0.072	-0.044	0.072
Next segment: Silence	**0.523**	**0.044** ***	**0.521**	**0.044** ***	**0.525**	**0.044** ***
Previous segment: Liquid	-0.052	0.046	-0.042	0.049	-0.040	0.050
Previous segment: Nasal	0.023	0.091	0.073	0.087	0.104	0.086
Previous segment: Plosive	0.262	0.286	0.210	0.290	0.242	0.289
Word stress: Stressed	-0.070	0.077	-0.108	0.070	-0.117	0.071
Number of syllables	-0.031	0.032	-0.020	0.032	-0.010	0.031
Lexical Neighbours	-0.031	0.022	0.027	0.022	0.029	0.022
Probability from prev. word	-0.014	0.017	-0.012	0.018	-0.016	0.017
Probability from next word	**0.038**	**0.018** *	**0.043**	**0.018** *	**0.038**	**0.017** *
Recently mentioned: True	-0.038	0.052	-0.041	0.052	-0.037	0.052
Register: Stories	**0.159**	**0.039** ***	**0.150**	**0.039** ***	**0.151**	**0.039** ***
Register: News	-0.057	0.066	-0.072	0.067	-0.061	0.066
-s Bias	-0.000	0.010				
Plural Dominance	**-0.023**	**0.011** *				
-s Bias : Plural Dominance	**0.014**	**0.006** *				
-s Frequency			-0.020	0.013		
Relative -s Frequency					-0.019	0.018
Random Effects	Variance	Standard Deviation	Variance	Standard Deviation	Variance	Standard Deviation
Noun (Intercept)	0.002	0.045	0.004	0.061	0.004	0.064
Speaker (Intercept)	0.011	0.106	0.011	0.105	0.011	0.106
Residual	0.130	0.360	0.130	0.360	0.130	0.360
Akaike Information Criterion	690.903		697.859		700.381	
Marginal R^2	0.493		0.479		0.476	
Conditional R^2	0.571		0.570		0.570	

REFERENCES

Bartoń, K. (2020). *MuMIn: Multi-Model Inference*. R package version 1.43.17

de Andrade Moral, R., Hinde, J., and Garcia Borges Demétrio, C. (2017). Half-normal plots and overdispersed models in r: The hnp package. *Journal of Statistical Software* 81, 1–23

Keuleers, E. and Daelemans, W. (2007). Memory-based learning models of inflectional morphology: A methodological case-study. *Lingue e linguaggio* 6, 151–174
